# Genome-Wide Association Mapping of Barley Yellow Dwarf Virus Tolerance in Spring Oat (*Avena sativa* L.)

**DOI:** 10.1371/journal.pone.0155376

**Published:** 2016-05-13

**Authors:** Bradley J. Foresman, Rebekah E. Oliver, Eric W. Jackson, Shiaoman Chao, Marcio P. Arruda, Frederic L. Kolb

**Affiliations:** 1 Department of Crop Sciences, University of Illinois at Urbana-Champaign, Urbana, Illinois, United States of America; 2 Department of Plant Sciences, North Dakota State University, Fargo, North Dakota, United States of America; 3 General Mills Crop Bioscience, Manhattan, Kansas, United States of America; 4 USDA-ARS Cereal Crops Research Unit, Fargo, North Dakota, United States of America; Julius Kuehn-Institute (JKI), GERMANY

## Abstract

Barley yellow dwarf viruses (BYDVs) are responsible for the disease barley yellow dwarf (BYD) and affect many cereals including oat (*Avena sativa* L.). Until recently, the molecular marker technology in oat has not allowed for many marker-trait association studies to determine the genetic mechanisms for tolerance. A genome-wide association study (GWAS) was performed on 428 spring oat lines using a recently developed high-density oat single nucleotide polymorphism (SNP) array as well as a SNP-based consensus map. Marker-trait associations were performed using a Q-K mixed model approach to control for population structure and relatedness. Six significant SNP-trait associations representing two QTL were found on chromosomes 3C (Mrg17) and 18D (Mrg04). This is the first report of BYDV tolerance QTL on chromosome 3C (Mrg17) and 18D (Mrg04). Haplotypes using the two QTL were evaluated and distinct classes for tolerance were identified based on the number of favorable alleles. A large number of lines carrying both favorable alleles were observed in the panel.

## Introduction

Oat (*Avena sativa* L.) is an important cereal crop grown worldwide with nutritional benefits for both livestock and humans [1]. Barley yellow dwarf (BYD) is one of the most destructive viral diseases of small grains. The disease was first described in barley by Oswald and Houston in 1951 [2] and affects all major cereal crops (rice, maize, wheat, oat and rye) as well as other grass species. The disease is caused by a group of phloem limited luteoviruses known as barley yellow dwarf viruses (BYDVs) that are obligately transmitted via aphid vectors [3]. Symptoms on oat depend on cultivar and environment but normally include leaf discoloration, stunted growth and blasting of florets. Economic losses due to BYDVs on oat range from 13–25 kg/ha for each 1% increase in incidence [4]. Methods of control for BYDVs include insecticides to control aphid populations; however, insecticides may only be feasible in highly intensive agricultural systems. The most effective way to control BYD is by planting tolerant cultivars [5].

There are several virus strains and at least 25 aphid species that transmit BYDV. Specific strains are classified into two genera based on genome structure. BYDVs PAV (*Rhopalosiphum padi* and *Sitobion avenae*), MAV (Sitobion *avenae*) [6], and SGV (*Schizaphis graminum*) make up the genus *Luteovirus*. *Cereal yellow dwarf virus* RPV [7] (*Rhopalosiphum padi;* formally known as *barley yellow dwarf virus* RPV) and *Maize yellow dwarf virus* RMV (*Rhopalosiphum maidis*) are in the genus *Polerovirus*. Viruses in the genera *Luteovirus* have replication-related protein related to members of *Tombusviridae*, while viruses in the genus *Polerovirus* have replication-related proteins similar to those of *Sobemoviruses* [8]. The most common vector in the United States is the aphid genus *Rhopalosiphum*, however, *Sitobion avenae* and *Schizaphis graminum* are commonly found in specific regions [9]. Rhopalosiphum is primarily holocyclic and prefers damp environments. There are two main species of *Rhopalosiphum* that vector BYDV, *Rhopalosiphum maidis* and *Rhopalosiphum padi*. *Rhopalosiphum padi* is the primary vector for BYDV in the Illinois, transmitting the PAV strain [10].

Two types of resistance to BYDV have been distinguished: virus resistance and field resistance. Field resistance is usually referred to as tolerance [11]. Virus resistance refers to low virus titer in infected plants whereas field resistance (tolerance) refers to the reduction of symptoms of infection independent of the virus titer. In this paper, resistance will be defined as reduced viral replication in infected plants [12]. Tolerance will therefore be defined as the development of mild or negligible symptoms in infected plants. It can also be stated as the ability of plants to yield under BYDV infection.

Historically, molecular marker technology in hexaploid oat has lagged behind that of maize, soybeans, and other diploid crops. Therefore, phenotyping for BYDV has been the only reliable method for screening breeding material for tolerance. Marker assisted breeding (MAB) would allow for more efficient selection of tolerant lines by aiding in the introgression of multiple genes controlling the trait. Until recently, the only available marker platforms in oat were restriction fragment length polymorphism (RFLP) [13], amplified fragment length polymorphism (AFLP) [14], and random amplified polymorphic DNA (RAPD) markers [15]. As molecular technology has improved, more and less expensive options have become available to oat breeders. Advancements in abundance and coverage with SSR markers have increased in oat [16–19] and the development of DArT markers [20], has provided another option for a high-throughput assay, as has genotyping-by-sequencing (GBS), although GBS is still new to oat [21]. Single nucleotide polymorphism (SNP) technology has also been developed for oat from expressed sequence tag (EST) information for SNP genotyping [22] and a high-throughput 6K oat SNP array has been developed [23].

Breeding for host-plant tolerance to BYD is the most effective method used to combat the destructive nature of the disease. It is believed that two to four genes are responsible for BYDV tolerance in oats [24]. Four QTL (BYDq1, BYDq2, BYDq3 and BYDq4) for BYD tolerance were identified [25] and were found on linkage groups OM1, 5, 7 and 24. Several other chromosomal regions with BYDV tolerance QTL have been identified in other studies. Barbosa-Neto et al. [26] in 2000 found 21 chromosomal regions distributed over 16 linkage groups that were associated with tolerance to BYD in oat. Using the previously mentioned 6K high density oat SNP array, several QTL were identified in two bi-parental populations. Two large effect QTL were identified on chromosome 3C (Mrg17) and 19A (Mrg04) [27].

BYDV tolerance has been examined in other crops such as wheat (*Triticum aestivum* L.), and barley (*Hordeum vulgare* L.). In wheat, two genes have been identified for tolerance, *Bdv1* and *Bdv2* [28–29]. However, these genes have not shown to be extremely useful under field test conditions [11]. Several genes and QTL have been identified in barley, and include *Ryd1* [30], *Ryd2* [31],*Ryd3* [32], and *Ryd4* [33]. Several other QTL have been identified in barley, including regions near *Ryd2* gene on chromosome 3HL [34]. Marker –assisted selection has been used in barley for the identification of *Ryd2* [35].

Genome-wide association studies (GWAS) provide a powerful approach to determine the genetic structure of complex traits in crops, and to date, GWAS have not been conducted for BYDV tolerance in oat. GWAS detect marker-trait associations by exploiting linkage disequilibrium between a marker allele and the causative QTL allele; however, population structure within the panel of lines and genetic relationships within the population can lead to false positive associations and therefore need to be taken into consideration [36, 37]. GWAS with population structure control have been successfully used to detect marker-trait associations for beta-glucan concentration in oat [38, 39]. Marker-trait associations that are identified will enable breeders to use marker assisted selection as well as genomic selection models. Using a set of oat cultivars and breeding lines as a GWAS panel, our objectives were to (1) assess population structure in the GWAS panel, (2) identify markers associated with BYDV tolerance and (3) to examine haplotypes within the population using the identified QTL.

## Materials and Methods

### Plant material and disease assessment

The association panel was developed by the Collaborative Oat Research Enterprise (CORE). The CORE is made up of a group of research scientists from North America and several other countries worldwide. Lines submitted to the panel by oat breeders had a high level of diversity and originated from six countries (United States, Canada, Sweden, United Kingdom, Norway and Germany). A total of 428 spring oat lines were included in the final panel and phenotyped for BYDV tolerance.

Tolerance for BYDV was evaluated at the University of Illinois Crop Sciences Research and Education Center, Urbana, IL in 2010 and 2011. Two replications of hills were planted in a randomized complete block design with 15 seeds per hill in the BYDV nursery. When the seedlings were in the three leaf stage (approximately 20 days after planting), the hills were inoculated with viruliferous *Rhopalosiphum padi* (L.) carrying the Illinois isolate BYDV-PAV. This isolate is maintained at the University of Illinois at Urbana-Champaign by continuous aphid cultures grown on barley plants. One month prior to field inoculation, aphids were distributed onto a larger number of barley plants in order to increase aphid numbers to a level satisfactory for the number of hills to be inoculated. The aphids are then mixed with cornmeal in a 50/50 ratio to prevent aphids from sticking together or to the inoculation device while inoculating. Each 15 seed hill is then hand inoculated using a device containing a loading cell that equally measures out the same volume of aphid/cornmeal mixture (roughly 70 aphids per inoculation). The aphid/cornmeal mixture then falls down onto the hill via a plastic tube. The plants were then sprayed with insecticide (Cygon 2E^™^) after one week to kill the aphids. BYDV tolerance was evaluated after stem elongation was completed. Each hill is then scored using a scale from 0 to 9 with 0 being assigned to the most tolerant plants and 9 indicating the most sensitive [40, 41].

### Phenotypic data analysis

Best linear unbiased predictors (BLUPs) for BYDV tolerance were calculated using a mixed model:

$$Y_{ijk}=\;\mu +year_{i}+block{\left(year\right)}_{ij}+line_{k}++{\left(year\;x\;line\right)}_{ik}+\varepsilon_{ijk}$$ (1)

Where *Y*_*ijk*_ is the observed BYDV phenotype, μ is the overall mean, *year*_*i*_ is the random effect of the *i*th year, *block*_*j*_ is the random effect of the *j*th block within the *i*th year, *line*_*k*_ is the random effect of the *k*th line, *year x line*_*ik*_ is the random effect of the interaction between the *i*th year and the *k*th line, and *ε*_*ijk*_ is the random error term. Broad-sense heritabilities on an entry-mean basis (H^2^) were calculated for BYDV tolerance using the variance components from the mixed model above (Eq 1) and equation (Eq 2):

$$H^{2}=\frac{\sigma_{g}^{2}}{\sigma_{e}^{2}/rt+\sigma_{ge}^{2}/t+\sigma_{g}^{2}}$$ (2)

where *H*^2^ is the entry-mean heritability, $\sigma_{g}^{2}$ is the genetic variance, $\sigma_{e}^{2}$ is experimental error, $\sigma_{ge}^{2}$ is the variance due to the interaction of genotype and environment, *r* is the number of replications, and *t* is the number of environments.

### Genotypic data

A total of 428 spring oat lines were included in the final panel and were genotyped using a high density oat SNP array containing 6000 SNPs. Genotyping was performed at the USDA-ARS Small Grains Genotyping Lab in Fargo, ND using the Infinium assay developed by Illumina [21]. A total of 2281 SNPs were identified to be polymorphic in the population. The marker number was further reduced by filtering for minor allele frequencies below 0.05 and markers with the proportion of missing genotypes greater than 0.10. Furthermore, by using the LDTagSNP Selection function in JMP Genomics 7 (SAS, Cary, NC) markers showing linkage disequilibrium (r^2^) higher than 0.8 were binned and a representative SNP from each bin was used [42]. This process was to help reduce the redundancy of markers explaining the same information and led to a final number of 1402 SNPs used in the association analysis.

A previously developed SNP-based physically anchored consensus map was used for marker locations [43]. The map was developed from 390 recombinant inbred lines from six bi-parental populations, which included 985 SNPs and 68 previously-published markers. The final map consisted of 21 chromosomes having a total map distance of 1838.8 cM. Updated chromosome nomenclature is shown in parentheses from a recently published consensus map [44].

### Genome-wide association analysis

Principal component analysis (PCA) was performed in order to examine the level of genetic structure in the panel (Q-matrix) via the PCA for Population Stratification function. The amount of relatedness (marker based kinship matrix, K-Matrix) was performed via the Relationship Matrix function. The above functions, as well as the marker-trait associations were performed in JMP Genomics 7 (SAS, Cary, NC). A Q-K mixed model was used for the marker-trait associations using the PCA values to form the Q-matrix, treated as fixed effect and the identical-by-descent (IBD) values for the K-matrix, treated as random effect. IBD values were calculated using equation (Eq 3);

$$IBD_{i,j}=\left(X_{i,l}-2p\right)*\left(X_{j,l}-2p\right)/2pq$$ (3)

Where *X* = 0,1,2 correspond to genotype BB, AB, AA at marker *l*, and p and q are the allele frequencies for allele A and B. The measure is averaged over all loci. The mixed model procedure was performed using the Q-K mixed model function with a false discovery rate α = 0.05 for multiple testing correction.

## Results

### Phenotypic data

A wide range of phenotypic variation was observed for BYDV tolerance (Table 1). This was expected due to the quantitative nature of the trait. The BYDV tolerance had a mean rating of 4.54 with maximum of 8.58 and a minimum of 1.33. High broad-sense heritability was observed for BYDV tolerance with a value of 0.91.

**Table 1:** Descriptive statistics and broad sense entry-mean heritability for BLUPs for BYDV tolerance in 428 spring oat lines.

	BLUPs					
	Mean	Min	Max	Range	SD^a^	H^2^^b^
BYDV	4.54	1.33	8.58	7.25	1.85	0.91

^a^ Standard deviation

^b^ Broad-sense heritability

### Genotypic data and population structure

The level of population structure was examined to gain understanding of the possible effect on the association analysis. Principal component analysis (PCA) using eigenvalues on marker data showed that PCA 1 accounted for 8.7% of the variation in the data. The first five PCAs accounted for a total of 23.3% of the variation in the data showing there may be some slight population structure in the panel of lines. Five principle components were determined to be sufficient and used in the final model. Relatedness was estimated using IBD statistics.

### Marker-trait associations for BYDV Tolerance

**Fig 1:**
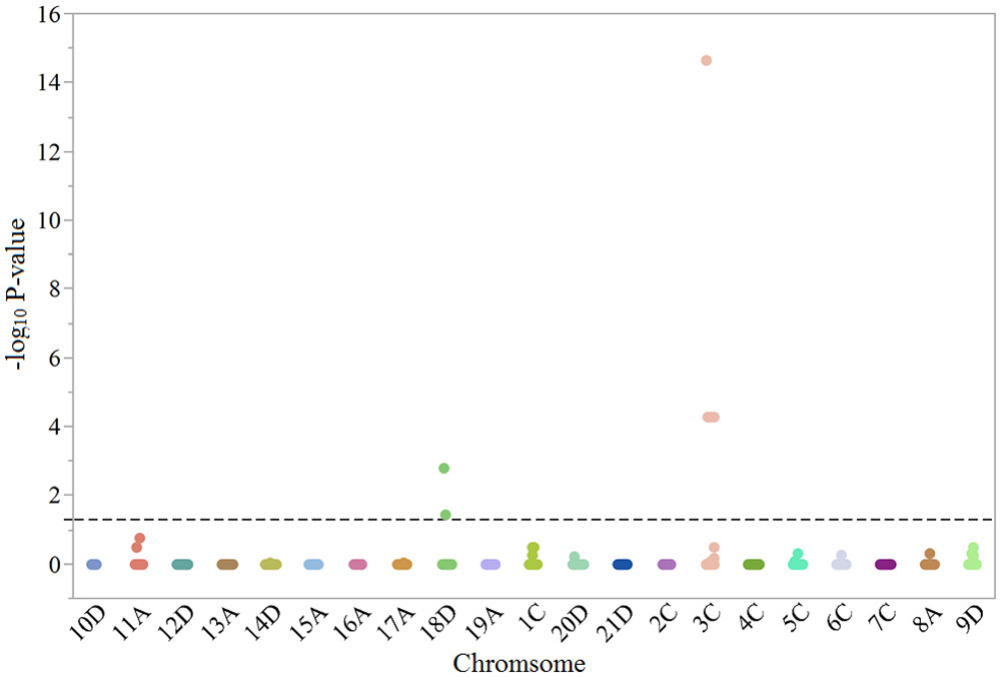
Genome-wide association for oat BYDV tolerance. The dotted line represents the significance threshold at α = 0.05 (-log_10_(p-value) ≥1.30), for false discovery rate adjusted P-values.

A total of six SNPs on chromosome 3C (Mrg17) and chromosome 18D (Mrg04) were significantly associated with BYDV tolerance (Fig 1). Note that in the most recent oat consensus map chromosome 3C is designated by Mrg17 and chromosome 18D is designated by Mrg04 [44]. SNP GMI_ES22_c20081_313 on chromosome 3C (Mrg17) accounted for 17% of the variance with an effect of -0.82 per favorable allele (Table 2). There were three other markers that were significant on chromosome 3C (Mrg17) that explained between 6% and 7% of the variation and had effects between -0.49 and -0.31. On chromosome 18D (Mrg04), SNP GMI_ES05_c3073_282 had the highest significance and explained 6% of the variation with an effect of 0.50. One other marker, SNP EMI_ES17_c6498_89, was also significant and explained 3% of the variation with an effect of -0.31.

**Table 2:** SNPs associated with BYDV tolerance in a panel of 428 spring oat lines, chromosomal position, *p*-values and marker effects.

SNP	C	cM	*p*^a^	r^2^	Adj. *p*^b^	Effect
GMI_ES22_c20350_257	3C (Mrg17)	113.2	6.84	0.07	4.29	-0.49
GMI_ES22_c20081_313	3C (Mrg17)	114.5	17.8	0.17	14.6	0.82
GMI_DS_LB_10400	3C (Mrg17)	115.1	6.91	0.06	4.29	-0.49
GMI_DS_CC1800_254	3C (Mrg17)	115.1	6.93	0.07	4.29	-0.31
GMI_ES17_c6498_89	18D (Mrg04)	132.7	3.82	0.03	1.45	-0.31
GMI_ES05_c3073_282	18D (Mrg04)	147.7	5.25	0.06	2.8	0.50

^a^ *p*-value reported on a -log_10_ scale;

^b^ False Discovery rate adjusted *p*-value

**Fig 2:**
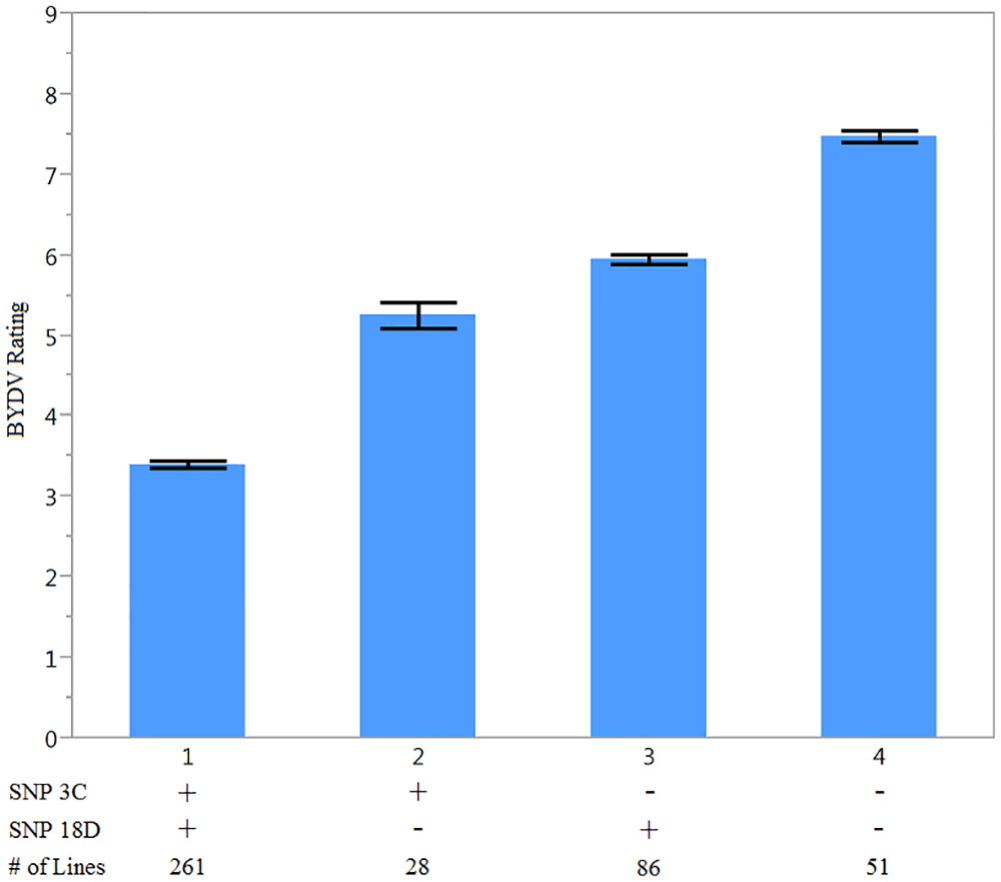
Phenotypic mean values for BYDV tolerance for oat haplotypes containing different SNP allele combinations with error bars representing one standard error. “+” signifies a favorable allele. BYDV rating scale is from 0 to 9 where 0 = most tolerant and 9 = most sensitive.

Using the most significant marker on chromosome 3C (Mrg17, GMI_ES22_c20081_313) and 18D (Mrg04,GMI_ES05_c3073_282), haplotypes were compared using contrasts (Table 3). For ease of use, the two SNP loci will be referred to SNP3C and SNP18D moving forward. Two entries were not used in the haplotype analysis due to missing data at one of the two markers. All other entries fell into the four possible haplotypes (haplotypes 1 through 4) (Fig 2). Haplotype 1 contained the favorable allele (“+ +”) at both loci and exhibited the lowest mean for BYDV tolerance (3.41). Haplotype 4 was constituted by having both unfavorable alleles (“- -”) at SNP3C and SNP18D. This haplotype displayed the highest mean BYDV tolerance at 7.46. Haplotypes 2 (favorable allele at SNP3C and unfavorable allele at SNP18D, “+ -”) and haplotype 3 (unfavorable allele at SNP3C and the favorable allele at SNP18D, “- +”) both had means that fell in between haplotype 1 and 4 (5.23 and 5.96). Contrasts between the four haplotypes were all significant at α = 0.05.

**Table 3:** Contrasts between haplotypes for BYDV tolerance in oat.

Haplotype	Estimate	Std. Error	F Ratio	prob > F
1 vs 2	-1.34	0.10	228.9	<.0001*
1 vs 3	-2.31	0.08	1183.7	<.0001*
1 vs 4	-3.69	0.08	1986.4	<.0001*
2 vs 3	-0.62	0.12	27.5	<.0001*
2 vs 4	-2.04	0.13	254.7	<.0001*
3 vs 4	-1.40	0.10	213.5	<.0001*

*Significance at α = 0.05

Two different sets of near-isogenic lines (NILs) were included in the panel from the University of Illinois at Urbana-Champaign [45]. Each set contains four lines and were developed using phenotypic selection. NIL family IL2250 contained four lines that ranged in BYDV rating from 3.14 to 6.98 (Table 4). NIL family IL2294 exhibited a similar range of BYDV ratings between 3.37 and 6.98.

**Table 4:** Barley yellow dwarf virus haplotypes for SNP3C and SNP18D in two sets of near-isogenic lines from Kolb et al. 2006.

NIL	Pedigree	GWAS BLUP	2 Year Mean (Kolb et al. 2006)	SNP3C	SNP18D	Haplotype
IL2250-18	Clintland 64*5 /IL86-5698	3.14	3.3	+	+	++
IL2250-14	Clintland 64*5 /IL86-5698	3.37	3.6	+	+	++
IL2250-3	Clintland 64*5 /IL86-5698	4.95	6.3	-	+	-+
IL2250-15	Clintland 64*5 /IL86-5698	6.98	8.6	-	-	—
IL2294-3	Clintland 64*5 /IL86-6404	3.37	4.5	+	+	++
IL2294-8	Clintland 64*5 /IL86-6404	4.73	3.6	+	+	++
IL2294-1	Clintland 64*5 /IL86-6404	6.31	8.9	+	-	+-
IL2294-2	Clintland 64*5 /IL86-6404	6.98	8.3	-	-	—

The haplotypes using SNP3C and SNP18D exhibited similar patterns in both families. In family IL2250, the two tolerant lines (IL2250-18 and IL2250-14) both had BYDV BLUPs of 3.14 and 3.37 respectively and both lines had a “+ +” haplotype. The moderately tolerant line IL2250-3 had BLUP of 4.95 with a haplotype of “- +” and the sensitive line IL2250-15 with a BLUP of 6.98 had the unfavorable allele at both SNPs (“- -“).

In the second family, the two tolerant lines IL2294-3 and IL2294-8 had BLUPs of 3.37 and 4.73. Both lines had the favorable allele at both SNPs (“+ +”). IL2294-1 had a BLUP of 6.31 and had the favorable allele at SNP3C and the unfavorable allele at SNP18D (“+ -“). IL2294-2 was the most sensitive line in the family and had a BLUP of 6.98. As expected this line had the unfavorable allele at both SNP markers (“- -“).

## Discussion

Barley yellow dwarf is one of the most destructive diseases of cereal crops worldwide. New breeding strategies such as marker-assisted selection and genomic selection along with high-throughput genotyping platforms can help to provide breeders with the tools necessary to introgress tolerance into elite cultivars. The identification of QTL associated with BYDV tolerance is an important first step to understanding the genetics of the tolerance mechanisms. In this study, genome-wide association mapping was performed on a panel of 428 spring oat cultivars using a 6K oat SNP array. This panel of lines includes a diverse amount of oat germplasm from several countries and breeding programs. A total of 1402 SNPs were used for genome-wide association for BYDV tolerance. A broad and continuous distribution was observed for BYDV tolerance across the panel and is in agreement with previous work that shows host plant tolerance for BYDV is multigenic [24–26, 46]. High heritabilities were observed which is consistent with recently observed heritability [27] but is higher than historical observations [25].

Population structure can result in false associations between markers and traits and therefore should be evaluated for proper analysis [47]. Principal component analysis with the SNP markers was used to determine the level of population structure in the panel. A moderate level of structure was observed via PCA analysis and is likely due multiple oat breeding programs in the CORE submitting lines. Relatedness (identical-by-descent) of lines was also evaluated for K-matrix calculation. To control false positive associations due to a high number of lines submitted from each program, the marker-trait association analysis was performed with a Q-K model containing both the matrices.

Previous studies have identified QTL for BYDV tolerance; however, many were performed with a very low number of markers, and the new SNP-based consensus maps did not use any of the markers reported before making it difficult to compare the linkage maps and the consensus map. Two bi-parental recombinant inbred lines populations using a previous version of the consensus map and the same 6K high density oat SNP array identified two large effect QTL that mapped to chromosomes 3C (Mrg17) and 19A (Mrg04) [21]. In this study, two QTL were identified on chromosome 3C (Mrg17) and 18D (Mrg04). These two QTL were located at 114.5 cM and 147.7 cM on their respective chromosomes (Table 2).

Several consensus map versions have been published to date, with the most recent in 2016. With the new map, nomenclature and some marker locations have changed from previous versions. Because of this, the new nomenclature has been added to the chromosome nomenclature of this study and previous studies. SNP GMI_ES05_c3073_282, representing the most significant QTL on chromosome 18D (Mrg04), did in fact agree with the QTL identified on chromosome 19A (Mrg04) from previously mentioned bi-parental studies [27]. For the SNP on chromosome 3C (Mrg17), the two studies identified a different significant marker but this is likely due to the LDTagSNP selection function that bins markers and tags a representative SNP for each bin. This study identified SNP GMI_ES22_c20081_313 while the identified SNP from the bi-parental studies was GMI_DS_CC1800_254. Linkage disequilibrium between the two markers in this study was 0.71, which fell just below the 0.80 threshold for the LDtagSNP function. It is also important to identify that the two tolerant parents (IL 86–1156 and IL 86–6404) and the susceptible parent (Clintland 64) from the bi-parental studies were included in this association panel. It appears that the two QTL identified in this GWAS study are in agreement with the results from the bi-parental studies.

The six significant marker sequences were blasted to various databases, including NCBI BLAST, BARLEX and the Wheat Genome Database. A 65% agreement was observed for the sequence with SNP3C for a mosaic virus helicase domain binding protein in wheat. Observing this was interesting since the protein was viral related and therefore could show conserved regions of viral tolerance/resistance genes. However, none of the identified BYDV genes/QTL in wheat or barley was identified in the searches.

Examining the potential haplotypes for the identified QTL showed that having the favorable alleles at both loci (“++”) leads to improved levels of tolerance (lower BYDV scores) (Fig 2). Although, all the contrasts comparing the four haplotypes were significant, classes of tolerance are visible based the number of beneficial alleles. From a plant breeding perspective, this is important because there are different implications when making selections. Identifying the most tolerant lines is important because this group can be selected to move forward in a breeding program or could be used as parents to improve other lines without tolerance. Haplotype 1 (“++”) is representative of this “high tolerance” class. The second group has medium levels of tolerance for BYDV. This class contains both haplotype 2 (“+-”) and haplotype 3 (“-+”). These classes contain cultivars with one positive allele. Even though there is a significant difference between haplotype 2 and 3, from a breeding perspective both haplotypes have medium levels of tolerance and therefore can be grouped together in the “moderate tolerance” class. The “sensitive” class is also important to be identified because these lines do not carry either BYDV tolerance QTL and could be discarded from a breeding program or if they contain other beneficial traits could be crossed with more tolerant lines to improve them.

From a breeding perspective, it is important to identify the tolerant lines that were submitted, as well as where they came from. The complete list of lines is included in the supplementary material as well as a breakdown of the haplotypes by breeding programs (S1 Table). Overall, 261 out of 428 lines included in the panel have the haplotype “+ +”, 114 lines contained one favorable allele and one unfavorable allele (“+ -”or “- +”) and 55 lines had two unfavorable alleles. The results appear to show that the oat community has effectively selected for BYDV tolerance. Combining BYDV tolerance with other beneficial traits will be important for future breeding.

The two families of NIL lines that were included in this study were both developed using phenotypic selection during the 1990s (Table 4). It is noteworthy that that the marker haplotype data agree with the phenotypically selected lines and a similar breakdown into distinct classes could be seen. Several other families of NILs were not included in the panel and therefore should also be genotyped in the future to further examine the identification of the high tolerance, moderately tolerant and sensitive groups.

## Conclusion

Host plant tolerance for BYD is the most effective mechanism for controlling the barley yellow dwarf virus. In this study, two QTL were identified on chromosomes 3C (Mrg17) and 18D (Mrg04) using a 6K high density oat SNP array and oat consensus map. This study also identified three main levels of BYDV tolerance based on the number of favorable alleles at the two loci. The largest group with over half the lines in the panel formed the “tolerant” class and had favorable alleles at both 3C (Mrg17) and 18D (Mrg04). Highly tolerant lines were observed from every program that made submissions to the panel, except from programs from Europe. The QTL and associated SNPs identified in this study can be used in marker-assisted selection or genomic selection programs to better improve host plant tolerance in oat for BYD.

## Supporting Information

S1 Table BYDV BLUPs, pedigrees and marker haplotypes for 428 oat lines. (XLSX)
